# 3.3 DISTURBANCES IN NEURAL OSCILLATIONS, GLUTAMATE, AND GABA: EFFECTS OF KETAMINE AND COMPARISON TO SCHIZOPHRENIA

**DOI:** 10.1093/schbul/sby014.005

**Published:** 2018-04-01

**Authors:** Lawrence Kegeles, Erin Stolz, Xiangling Mao, Najate Ojeil, Raphael Massuda, Mariana Pedrini, Maryam Bayatmokhtari, Mark Slifstein, Anissa Abi-Dargham, Matthew Milak, Carolyn Rodriguez, Chi-Ming Chen, Dikoma Shungu

**Affiliations:** 1 Columbia University & New York State Psychiatric Institute; 2 University of Connecticut, Storrs; 3 Weill Cornell Medical College; 4 Universidade Federal do Rio Grande do Sul; 5 Mount Sinai School of Medicine; 6 Stony Brook University School of Medicine; 7 Stanford University

## Abstract

**Background:**

In schizophrenia (SCH), proton MRS studies of the medial prefrontal cortex (MPFC) show elevated glutamine (Gln) or the combination of Glu and Gln (Glx) in unmedicated patients. Studies in healthy human subjects demonstrate ketamine-induced acute increases in MPFC Glu or Gln. Together, these findings raise the question of potential disturbances in excitation-inhibition balance in the illness, possibly arising from NMDA receptor deficits in GABAergic interneurons. We investigated these questions following acute ketamine administration by using repeated 15-minute MRS acquisitions of Glx and GABA with simultaneous EEG and comparing the results with the same modalities acquired in SCH.

**Methods:**

We enrolled 11 healthy volunteers (age 18–55) who were given a constant i.v. infusion of ketamine 0.5 mg/kg over 40 min during a combined EEG and 1H MRS study. Glx and GABA were acquired in the pregenual MPFC using a 3T GE system and a J-edited PRESS sequence. Sequential MRS acquisitions each of 15 min duration (90 min total) were obtained before, during, and following the infusion. EEG was recorded using an MRI-compatible 64-channel system with direct current BrainAmp MR amplifiers (Brain Products GmbH). Post-ketamine EEG data were analyzed in frontal electrodes for gamma and delta alterations. EEG and MRS data were also acquired in 12 patients with SCH with these systems.

**Results:**

Neurochemicals Glx and GABA showed acute increases within 15–30 minutes following the initiation of ketamine infusion, more pronounced for GABA (13% increase, p = .04 by paired t test). Gamma amplitude in left and right frontal electrodes increased in the first 15-minute average after initiation of ketamine (p < .05), with no evidence of earlier gamma decrease. Left delta amplitude decreased linearly following ketamine (p < .01). Peak GABA concentration correlated inversely with average left delta amplitude in the immediately subsequent 15-minute acquisition. Data in SCH showed similar elevations in GABA and gamma amplitude.

**Discussion:**

These data show the feasibility of attaining time resolution of Glx and GABA changes in the several-minutes range with standard PRESS J-edited 1H MRS, and simultaneous sub-second resolution with EEG. There were no indications in these frontal electrodes of very early GABAergic inhibition leading to disinhibition of Glx, which may occur in other brain regions following ketamine administration. The GABA, Glx, and EEG alterations found here following ketamine administration are consistent with stable alterations reported in unmedicated patients with SCH and are compatible with an NMDA receptor deficit mechanism in the illness. They show homeostatic rebalancing at elevated levels as found in SCH itself. Excitation-inhibition rebalancing at abnormally elevated levels may pose a risk of neuronal damage that persists in untreated psychosis.

